# Influence of dietary specialization and resource availability on geographical variation in abundance of butterflyfish

**DOI:** 10.1002/ece3.253

**Published:** 2012-07

**Authors:** Rebecca J Lawton, Morgan S Pratchett

**Affiliations:** ARC Centre of Excellence for Coral Reef Studies, James Cook UniversityTownsville QLD, 4811, Australia

**Keywords:** Chaetodontidae, coral reef, distribution, local abundance, niche breadth, vulnerability

## Abstract

Empirical evidence indicates that both niche breadth and resource availability are key drivers of a species’ local abundance patterns. However, most studies have considered the influence of either niche breath or resource availability in isolation, while it is the interactive effects that are likely to influence local abundance. We examined geographic variation in the feeding ecology and distribution of coral-feeding butterflyfish to determine the influence of dietary specialization and dietary resource availability on their local abundance. Dietary composition and abundance of five butterflyfish and coral dietary resource availability were determined at 45 sites across five locations (Lizard Island and Heron Island, Great Barrier Reef; Kimbe Bay, Papua New Guinea; Noumea, New Caledonia; and Moorea, French Polynesia). Multiple regression models using variables representative of total dietary resource availability, availability of specific dietary resources, and interspecific competition were used to determine the best predictors of local abundance across all sites and locations for each species. Factors influencing local abundance varied between butterflyfish with specialized and generalized diets. Dietary resource availability had the strongest influence on the abundance of *Chaetodon trifascialis*—the most specialized species. Local abundance of *C. trifascialis* was best predicted by availability of the *Acropora* corals that it preferentially feeds on. In contrast, abundance of generalist butterflyfish was poorly described by variation in availability of specific resources. Rather, indices of total dietary resource availability best predicted their abundance. Overall, multiple regression models only explained a small proportion of the variation in local abundance for all five species. Despite their relatively specialized diets, dietary resource availability has limited influence on the local abundance of butterflyfish. Only the most specialized species appear to be consistently limited by prey availability. Local and total abundance of species are influenced by a wide range of different factors and there is definite need to conduct independent species assessments.

## Introduction

Many macroecological studies have sought to understand what determines a species’ patterns of abundance (Brown 1984; Gregory and Gaston 2000). Niche breadth is often invoked as a key determinant following Brown's (1984) niche breadth hypothesis, which predicts that generalist species will be locally abundant as they are able to exploit a wide range of environmental conditions and resources, while specialist species will be locally rare (Brown 1984; Brown et al. 1995). However, the link between niche breadth and abundance remains unclear, with studies both supporting (Pyron 1999; Harcourt et al. 2002) and failing to find (Gaston et al. 1997; Gregory and Gaston 2000; Brandle et al. 2002) a relationship. These contrasting results may in part be due to resource availability—specialist species may attain high local abundance if their preferred resources are also locally abundant. Resource availability has been identified as a key determinant of local abundance patterns in multiple studies across a range of taxa (e.g., fish: Holbrook et al. 2000; birds: Tellería and Pérez-Tris 2003; mammals: Womble and Sigler 2006; bees: Roulston and Goodell 2011). But despite the recognized importance of both niche breadth and resource availability in determining local abundance (e.g., Munday 2002), most studies have considered these factors in isolation and the interactive effects between them remain largely unstudied.

Local abundance is likely to be influenced by both niche breadth and resource availability, such that at a given location, local abundance will be determined not only by the availability of resources but also by an organism's ability to utilize those resources. As generalist species are able to utilize a wide range of resources, their local abundance should be largely unaffected by differences in the availability of specific resources, as long as total resource availability remains constant. In contrast, specialist species are expected to be limited by the availability of critical resources (Brown 1984). Accordingly, their local abundance is more likely to be correlated with the abundance of the specific resources they specialize on, rather than total resource availability (Munday 2002; Pratchett and Berumen 2008), and will therefore be more variable than that of generalist species. Indirect evidence for these predictions comes from several sources. Specialist species are more sensitive to changes in resource availability (e.g., Harcourt et al. 2002; Kotze and O'Hara 2003; Swihart et al. 2003; Julliard et al. 2004; Aitken and Martin 2008) and can also have more restricted distributions across habitats with varying resource availability (Ostergard et al. 2009) compared to generalist counterparts. Furthermore, the abundance of some specialist species can vary significantly between sites with similar levels of total resource availability, but differing availability of specific resources (Graham 2007). These findings suggest that overall indices of resource availability will not provide an accurate reflection of resource availability for specialist species, and as such, these indices may not be the best predictors of their local abundance. However, the relative importance of total resource availability versus the availability of specific resources as determinants of local abundance for specialist species has rarely been examined.

Butterflyfish (genus *Chaetodon*) provide an ideal model group to investigate the influence of resource availability on the local abundance of specialist versus generalist species. The dietary composition and level of dietary specialization of species within this genus vary considerably, ranging from generalist species such as *Chaetodon citrinellus*, which feed on a broad range of hard coral species as well as soft corals and other reef macroinvertebrates, to highly specialized species such as *C. trifascialis*, which feed on only a small number of hard coral species (Pratchett 2005; Lawton et al. 2012, In press). The composition and abundance of hard coral species, the key dietary resource of these fish, also vary markedly at both a local (e.g., between reefs within a single location) and geographical scale (Edmunds and Bruno 1996; Veron 2000; Berumen et al. 2005). There is also evidence that the local abundance of coral-feeding butterflyfish is related to the availability of coral dietary resources. Numerous studies have found a strong link between total hard coral cover and butterflyfish abundance (Bell and Galzin 1984; Bouchon-Navaro et al. 1985; Cadoret et al. 1999; Bozec et al. 2005; Pratchett and Berumen 2008; Emslie et al. 2010, but see Bell et al. 1985; Fowler 1990) and many butterflyfish have also been observed to decline in abundance following coral loss (e.g., Sano et al. 1987; McClanahan et al. 2002; Halford et al. 2004; Pratchett et al. 2006; Graham 2007). Furthermore, predation on coral-feeding butterflyfish is generally thought to be very low (Cole et al. 2008) and their local abundance tends to be fairly stable in the absence of changes in coral availability (Halford et al. 2004), implying that availability of coral dietary resources, rather than predation or recruitment-driven processes, is likely to be a primary driver of local abundance patterns for these fish. Empirical data indicate that butterflyfish with specialized diets are more susceptible to coral loss than generalist feeders (Pratchett et al. 2006; 2008b; Graham 2007), suggesting that different factors are likely to determine the local abundance of specialist and generalist butterflyfish species. These characteristics make butterflyfish ideal candidates to explore the links between ecological versatility, resource availability, and local abundance.

Coral-feeding butterflyfish have been identified as one of the most vulnerable groups of reef fish to the combined effects of ongoing global coral loss and degradation of coral reef habitats (Wilson et al. 2006; Pratchett et al. 2008b). Identifying key drivers of their local abundance has important implications for understanding how butterflyfish are likely to be impacted by climate change. Many species preferentially feed on corals from the genus *Acropora* (Pratchett 2005; Cole et al. 2008), which are themselves vulnerable to a range of disturbances on reefs (Marshall and Baird 2000; Madin and Connolly 2006; Pratchett 2010). If local abundance of butterflyfish, particularly specialist species, is also linked to these corals, then their vulnerability to global climate change is likely to be greatly increased. Although variation in butterflyfish abundance and the role of hard coral cover as a driver of local abundance patterns have been previously investigated at a number of spatial scales (e.g., geographic regions: Findley and Findley 2001; reefs: Bozec et al. 2005; physiognomic reef zones: Pratchett and Berumen 2008), the influence of specific dietary resources on abundance patterns is yet to be examined. Comparisons of local abundance and coral resource availability at replicate sites within different locations will help identify the spatial scale at which these factors are influencing populations (Underwood and Chapman 1996; Hughes et al. 1999; Munday 2002).

Although a wide range of factors can potentially influence the abundance of reef fish, we focus here on the influence of coral dietary resources due to the strong reliance of many butterflyfish on corals, which are highly vulnerable to the impacts of global climate change. Therefore, the objective of this study was to investigate the influence of dietary specialization and coral dietary resource availability on the local abundance of butterflyfish. Our specific aims were to (1) determine the dietary composition, level of dietary specialization, and local abundance of five butterflyfish across five geographically separated locations; (2) compare the local abundance of each species to the availability of dietary resources at each location; and (3) determine the best predictors of local abundance for each species across all locations. We expected that local abundance of dietary specialists would be best predicted by the availability of their preferred coral resources, whereas the local abundance of dietary generalists would be best predicted by total dietary resource availability. Local abundance of butterflyfish may also be modified by interspecific competition and the presence of other butterflyfish species. Butterflyfish are known to aggressively defend territories and dominant competitors have been observed to restrict the access of subordinates to habitats containing preferred corals (Crosby and Reese 2005; Berumen and Pratchett 2006a). Therefore, the influence of interspecific competition was also considered as a possible determinant of local abundance.

## Methods

### Study sites and species

**Figure 1:**
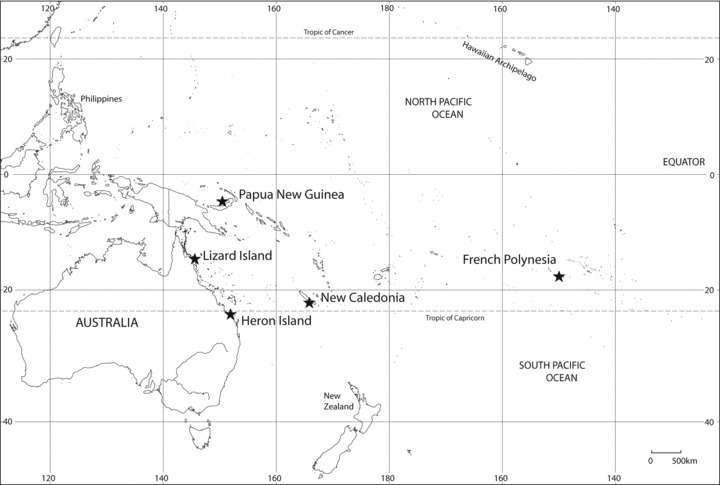
Map of the five locations sampled in this study. Abbreviations used throughout this paper are indicated for each location. Heron Island, Great Barrier Reef (HI); Lizard Island, Great Barrier Reef (LI); Kimbe Bay, Papua New Guinea (PNG); Noumea, New Caledonia (NC); and Moorea, French Polynesia (FP).

This study investigated local abundance patterns of five common and widespread species of butterflyfish—*C. auriga*, *C. vagabundus*, *C. citrinellus*, *C. lunulatus*, and *C. trifascialis*. *Chaetodon auriga*, *C. vagabundus*, and *C. citrinellus* are all facultative corallivores—consuming hard corals as well as other small motile invertebrates and soft corals; while *C. lunulatus* and *C. trifascialis* are both obligate corallivores, feeding almost exclusively on hard corals (Pratchett 2005; Cole et al. 2008; Lawton et al. In press). Sampling was conducted at five geographically separated locations throughout the Pacific: (1) Lizard Island, Northern Great Barrier Reef; (2) Heron Island, Southern Great Barrier Reef; (3) Kimbe Bay, Papua New Guinea; (4) Noumea, New Caledonia; (5) Moorea, French Polynesia (Fig. 1). These sites are separated by 1100–6600 km and distributed along known diversity gradients (Bellwood and Hughes 2001). At each location, nine distinct sites across a range of habitats (e.g., exposed front reef, sheltered back reef, fringing reef, shallow water patch reef) were sampled to determine butterflyfish abundance. At three of these sites, feeding observations were conducted to determine the dietary composition of the five focal species in each location. To maintain consistency between varying habitats and locations, only the rest crest zone was sa-mpled for both abundance surveys and feeding observations.

### Butterflyfish abundance and coral composition

The abundance of butterflyfish (focal species as well as all other congenerics) was determined using underwater visual census. At each site, 50 × 4 m belt transects were laid parallel to the reef crest. Transects were delineated using a 50-m fibreglass tape and the number of individual butterflyfish >50 mm total length located within 2 m of either side of the tape was recorded to species level. A total of five replicate transects were surveyed at each site, giving a total of 225 transects across all locations. To allow butterflyfish abundance to be directly related to coral cover and dietary resource availability at each site, coral composition on each of the five transects used to census butterflyfish abundance was determined using 50-m point intercept transects. Along each transect, the substrate directly beneath 200 uniform sampling points (spaced every 25 cm) was recorded to species level for hard (scleractinian) corals, and to broad categories for all other substrate types (e.g., noncoral substrate, macroalgae). To provide an index of coral species richness, the total number of different hard coral species detected on each individual transect was determined. The Shannon–Wiener J’ index was calculated for each individual transect following Zar (1999) as an index of coral species diversity.

### Dietary composition

Field observations of feeding behavior at three sites in each location were conducted to determine the dietary composition of each of the five focal species. Individual adult butterflyfish were randomly selected and followed at a distance of 2–5 m for 3 min, following Pratchett (2005). Every effort was made to ensure individual fish were not observed more than once. At least 20 observations for each species were conducted throughout the day at each site, giving a total of at least 60 observations at each location for each species. During each observation, the total number of bites taken on each of six coral taxa groupings (*Acropora*, *Montipora*, *Pocillopora*, *Porites*, Favidae, other hard corals), noncoral substrates (e.g. sand, rubble, pavement) and any other items (e.g., algae, noncoral macroinvertebrates) was recorded, following Pratchett (2005). Smith's measure of niche breadth (*FT*) was used to determine the relative degree of dietary specialization for each species at each site (Smith 1982). This measure takes into account resource availability and is less sensitive to the use of rare resources compared to other niche breadth measures (Krebs 1999). *FT* is a standardized measure, ranging from 0 (most specialized) to 1 (least specialized), therefore allowing comparison of the level of specialization between sites, locations, and species.

### Statistical analyses

Variation in the abundance of the five focal butterflyfish species and coral assemblage composition among locations and sites was assessed using multivariate analyses of variance (MANOVAs) comparing the mean abundance of butterflyfish and mean percent cover of corals (grouped into six taxa, plus noncoral substrates and other items) among nine sites nested within each of the five geographically separated locations. Abundance data were log_10_ transformed and coral cover data were arc-sine transformed to satisfy assumptions of multi-variate homogeneity and normality. Pillai's trace statistic was used to determine the significance of MANOVA results. Where significant, variation in butterflyfish abundance and coral composition at each site were displayed using canonical discriminant analyses (CDA). To assist with interpretation of the CDA, structural coefficients of the butterflyfish species and coral taxa were plotted as vectors to indicate the predominant species and taxa at each site. To explore inter-specific differences in the relative importance of different spatial scales of comparison, variance in the abundance of each focal species was partitioned among locations, sites, and transects, using the mean squares ratios of univariate *F* values from independent nested ANOVAs. To explore the relationship between niche breadth and local abundance, the average niche breadth of each species was calculated for each location and plotted against the average abundance of each species in each location. Due to the small number of datapoints, a formal quantitative analysis of this relationship was not undertaken.

Separate multiple linear regressions using a forward stepwise method were run for each of the five focal species to identify factors significantly contributing to their local abundance. Data collected on each transect were treated as an individual replicate. An initial multiple regression analysis was run for each species with their abundance as the dependent variable and a standard set of predictor variables entered into the model simultaneously. Predictor variables with nonsignificant beta coefficients in this initial model were discarded. The model was then rerun using the remaining predictor variables that were entered hierarchically in order of their decreasing contribution to the initial model. Only predictor variables resulting in a significant change in the *R*^2^ value on this second model (based on an *F*-ratio test conducted in SPSS) were retained and used in the final model. Residual plots, homogeneity tests, and the Durbin–Watson test were used to ensure assumptions were met. Predictor variables used for each species in the initial model were as follows: hard coral species richness, hard coral species diversity, percent total hard coral cover, the percent cover of any dietary category comprising more than 1% of total diet across all locations, and the ratio of total hard coral cover to total abundance of all congeneric butterflyfish. Predictor variables were chosen to be representative of the influence of total dietary resource availability (coral species richness and diversity indices, percent total hard coral cover), availability of specific dietary resources (percent cover of dietary categories), and interspecific competition (ratio of total coral cover to total abundance of all congeneric butterflyfish). Predictor variables used for each species in each stage of the analysis are given in Table 1.

**Table 1:** Predictor variables used in multiple regression analyses for five species of butterflyfish

Species	Dietary categories	Significant variables	Final model
*C. auriga*	Noncoral substrate, *Acropora*, other hard corals, other	*Acropora*	–1
*C. vagabundus*	Noncoral substrate, *Acropora*, *Pocillopora*, *Montipora*, *Porites*, other	*Pocillopora*, hard coral species diversity	*Pocillopora*, hard coral species diversity
*C. citrinellus*	Noncoral substrate, *Acropora*, *Pocillopora*, *Montipora*, *Porites*, Favidae, other hard corals, other	Number of hard coral species, hard coral species diversity, total coral cover/abundance congenerics	Number of hard coral species, total coral cover/abundance congenerics
*C. lunulatus*	Noncoral substrate, *Acropora*, *Pocillopora*, *Montipora*, *Porites*, Favidae, other hard corals	Noncoral substrate, total coral cover/abundance congenerics, number coral species	Noncoral substrate, total coral cover/abundance congenerics, number coral species
*C. trifascialis*	*Acropora*, *Pocillopora*, *Montipora*	*Acropora*, total coral cover/abundance congenerics, *Montipora*	*Acropora*, total coral cover/abundance congenerics

1 Final model was not significant.

Data displayed are any dietary items comprising more than 1% of total diet across all locations (dietary categories), significant predictor variables in the initial model (significant variables), and predictor variables included in the final model (final model). Significant variables are listed in order of their decreasing contribution to the initial model. See Methods section for more details.

## Results

### Butterflyfish assemblages

**Figure 2:**
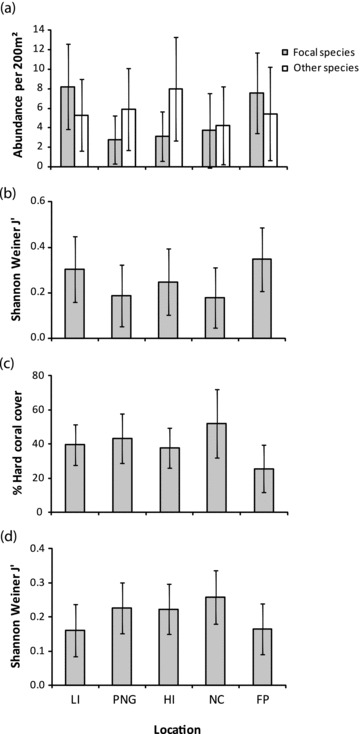
Characteristics of butterflyfish assemblages and coral communities at five locations. (a) Mean (±SE) abundance per transect of focal species and other butterflyfish species; (b) mean (±SE) species diversity (Shannon–Weiner J’ index) of all butterflyfish; (c) mean (±SE) percent hard coral cover; and (d) mean (±SE) species diversity (Shannon–Weiner J’ index) of hard corals. Location abbreviations follow Figure 1.

**Figure 3:**
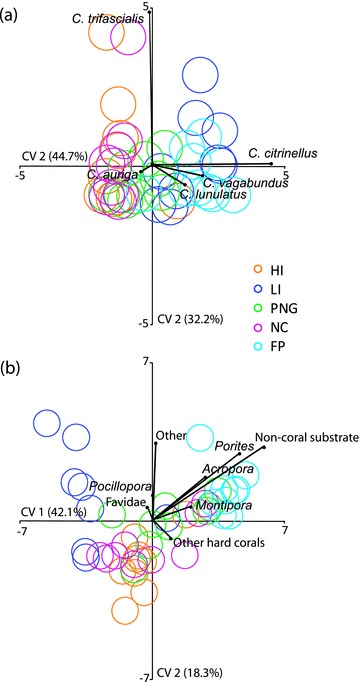
Canonical discriminant analyses of (a) butterflyfish communities and (b) coral assemblages at nine sites in each of five locations (HI, LI, PNG, NC, FP). Location abbreviations follow Figure 1. Vectors are structural coefficients indicating the relative abundance of butterflyfish species (a) and coral taxa (b).

**Figure 4:**
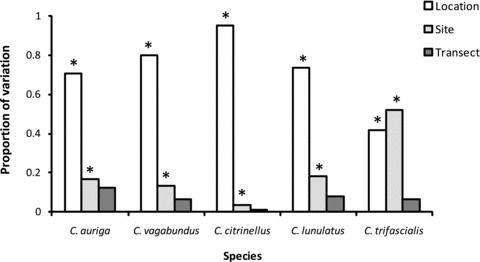
Proportion of variation in abundance of butterflyfish assemblages occurring among locations, sites, and transects.*P < 0.05 (ANOVA Bonferroni-corrected significance levels).

A total of 2440 individual butterflyfish were recorded across all 225 transects sampled, corresponding to an average of 10.8 (±0.4 SE) fish per transect. There was no consistent pattern with longitude in either abundance (Fig. 2a) or diversity (Fig. 2b). The five focal butterflyfish species accounted for 47% of the total number of butterflyfish recorded across all locations. The abundance of the five focal species was highest at Lizard Island (8.2 fish per transect ± 0.7 SE) and was lowest in Papua New Guinea (2.8 fish per transect ± 0.4 SE). Abundance of the five focal species varied significantly among locations (nested MANOVA, Pillai's trace = 1.019, df = 20,716, *P* < 0.001) and sites nested within locations (nested MANOVA, Pillai's trace = 1.8768, df = 200,900, *P* < 0.001). There was some partitioning of site centroids by location in the CDA; however, there was considerable overlap of site centroids from different locations, inferring that the butterflyfish assemblages were generally similar between sites and locations (Fig. 3a). Structural coefficients indicated that sites in French Polynesia and Lizard Island were characterized by a high abundance of *C. vagabundus* and *C. lunulatus* (Fig. 3a). Variance components indicated that for all species except *C. trifascialis*, most of the variation in abundance was attributable to variation among locations, rather than sites or transects. In contrast, variation in abundance was similar at both sites and locations for *C. trifascialis* (Fig. 4).

### Coral composition

Both total hard coral cover and hard coral species diversity (measured by the Shannon–Weiner J’ index) were highest in New Caledonia (coral cover: 53.2%± 2.9 SE, species diversity: 0.26 ± 0.01 SE), but the total number of different hard coral species recorded on each transect was highest in Papua New Guinea (18.1 species ± 0.8 SE) (Fig. 2c and d). Total hard coral cover, hard coral species diversity, and the number of hard coral species detected on each transect were all lowest in French Polynesia (coral cover: 25.5%± 2.0 SE, species diversity: 0.16 ± 0.01 SE, coral species detected: 4.7 ± 0.3 SE). Coral assemblage composition varied significantly between locations (nested MANOVA, Pillai's trace = 2.634, df = 32,704, *P* < 0.001) and sites nested within locations (nested MANOVA, Pillai's trace = 4.538, df = 320,1440, *P* < 0.001). The groupings of site centroids in the CDA indicated that coral assemblages at each location were more distinct than butterflyfish assemblages (Fig. 3b). Fewer site centroids from different locations overlapped with each other in the coral CDA and, in contrast to the butterflyfish CDA, centroids from Lizard Island and French Polynesia sites were completely separated from each other. Structural coefficients indicated that sites in French Polynesia were characterized by a high abundance of noncoral substrates, reflecting the low total hard coral cover at this location. Coral assemblages at Heron Island, New Caledonia, and Lizard Island were dominated by *Acropora* corals, with this taxon comprising more than 74%, 63%, and 56%, respectively, of the total hard coral cover at these locations. French Polynesia sites were dominated by *Porites* and *Montipora* corals—these two taxa accounting for >80% of the total coral cover in this location. In contrast, cover of different coral taxa was variable at sites in Papua New Guinea, with no one taxa dominating assemblages in this location.

### Dietary composition and specialization

**Figure 5:**
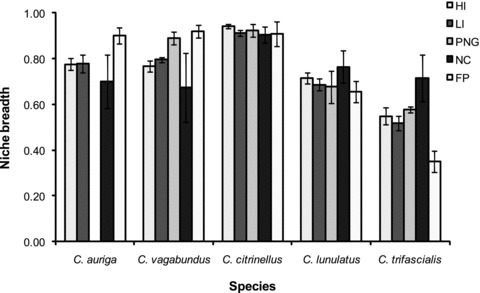
Mean (±SE) dietary niche breadth (*FT)* of five butterflyfish species at five locations. Location abbreviations follow Figure 1.

Feeding observations were completed for a total of 1506 individual fish (see Table A1). All butterflyfish studied fed from the surface of live corals, but the proportional feeding on corals versus noncoral substrates varied greatly. *Chaetodon auriga* took at least 85% of all bites on noncoral substrates at each location (see Table A1). *Chaetodon vagabundus* also fed predominantly on noncoral substrates, taking at least 90% of all bites on this category at Lizard Island, Heron Island, and French Polynesia, and more than 60% of all bites on this category in New Caledonia and Papua New Guinea, respectively (see Table A1). Hard corals contributed significantly to the diet of *C. vagabundus* in Papua New Guinea, accounting for 32.0% (±5.2 SE) of all bites, most of which were taken on *Montipora*, *Pocillipora*, and *Porites* corals. Both *C. auriga* and *C. vagabundus* had relatively high niche breaths (Fig. 5), indicating that they were both generalist feeders. *Chaetodon citrinellus* fed across a broad range of hard coral taxa, non-coral substrates, and other dietary items, and diet was variable among locations (see Table A1). Between 11% and 81% of all bites were taken on noncoral substrates at each site, with the majority of bites at New Caledonia and French Polynesia taken on this category (53.3 ± 9% S.E. and 60.4 ± 18% SE, respectively). Diet was dominated by *Acropora* corals at Heron Island, with roughly half of all bites on hard corals taken on this taxon, while at Lizard Island *Pocillopora* corals accounted for nearly 40% of all bites on hard corals on average. In contrast, in both New Caledonia and Papua New Guinea, bites were spread reasonably evenly between all hard coral resource categories. *Chaetodon citrinellus* was the most generalized of all species, with a high-dietary niche breadth in all locations (Fig. 5). *Chaetodon lunulatus* took at least 97% of all bites on hard corals at all locations (see Table A1). At each site, bites were spread across all hard coral resource categories, with the exception of French Polynesia where diet was dominated by *Montipora* and *Porites* corals (at least 80% of all bites). Niche breadth was reasonably high, indicating that *C. lunulatus* had a fairly generalized diet (Fig. 5). *Chaetodon trifascialis* took 100% of bites on hard corals at all locations (see Table A1) and had a much more specialized diet than the other species. Diet was dominated by *Acropora* corals (>90% of all bites) in all locations except French Polynesia where *Montipora* and *Pocillopora* corals were also fed on, albeit in relatively low proportions (<30% of bites). *Chaetodon trifascialis* was the most specialized of all species, with a low to moderate niche breadth in each location (Fig. 5).

### Niche breadth and local abundance

**Figure 6:**
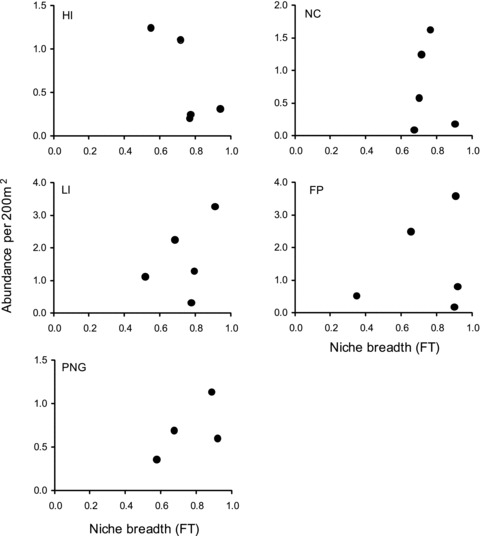
Average niche breadth (*FT*) and abundance per 200 m^2^ of five species of coral-feeding butterflyfish across five geographic locations. Location abbreviations follow Figure 1.

Plots of the average niche breadth and local abundance of each species at each of the five study locations were variable in pattern. Overall, there was no strong trend toward a positive or negative relationship between these factors (Fig. 6).

### Regression models

Multiple linear regression analyses indicated that the local abundance of each of the five focal species was explained by a different set of variables. Availability of specific resource categories were significant predictors of abundance for three of the study species (Tables 1 and 2), but not for *C. auriga* or *C. citrinellus*. In particular, the abundance of the dietary specialist *C. trifascialis* was best predicted by *Acropora* corals, its preferred coral prey (Table 2). The abundance of *C. vagabundus* was positively correlated with the availability of *Pocillopora* corals, while the abundance of *C. lunulatus* was negatively correlated with the availability of noncoral substrates (Table 2). Our index of interspecific competition (the ratio of total hard coral cover to total abundance of all congeneric butterflyfish) was negatively correlated with the abundance of *C. citrinellus*, *C. lunulatus*, and *C. trifascialis*, while indices of coral species richness and diversity were a significant predictor of abundance for *C. vagabundus*, *C. citrinellus*, and *C. lunulatus* (Table 2). Overall regression models and individual predictor variables were highly significant for all species except *C. auriga*, however, the models only explained a low proportion of the variation in abundance for each species, ranging from 11.5% (adjusted *R*^2^) for *C. vagabundus* to 20.8% for *C. trifascialis* (Table 3).

**Table 2:** Coefficients of multiple regression models for four species of butterflyfish

Species	Final predictors	*B*	SE *B*	β
*C. vagabundus*	Pocillopora	2.354	0.437	0.344***
	Hard coral diversity	−0.442	0.181	−0.156*
*C. citrinellus*	Number of coral species	−0.15	0.003	−0.280***
	Total coral cover/abundance congenerics	−1.201	0.292	−0.256***
*C. lunulatus*	Noncoral substrate	−0.394	0.086	−0.306***
	Total coral cover/abundance congenerics	−0.897	0.252	−0.226***
	Number coral species	−0.015	0.003	−0.320***
*C. trifascialis*	*Acropora*	0.590	0.077	0.485***
	Total coral cover/abundance congenerics	−0.769	0.201	−0.242***

The unstandardized beta coefficients (*B*), their standard errors (SE *B*), and the standardized beta coefficients (β) for the predictor variables included in the final regression model for each species are presented.*P < 0.05,**P < 0.01,***P < 0.001.

**Table 3:** Final multiple regression results for abundance of five species of butterflyfish

Species	Adjusted *R*^2^	Sum of Squares	df	Mean square	*F*	Significance
*C. auriga*	−0.004	0.00	1,224	0.000	0.020	<0.889
*C. vagabundus*	0.115	1.546	2,224	0.773	15.550	<0.001
*C. citrinellus*	0.163	4.194	2,224	2.097	22.751	<0.001
*C. lunulatus*	0.159	3.491	3,224	1.164	15.114	<0.001
*C. trifascialis*	0.208	3.212	2,224	1.606	30.340	<0.001

## Discussion

This study revealed that the factors influencing local abundance varied among butterflyfish, with pronounced differences between specialist versus generalist species. Dietary resource availability had the strongest influence on abundance patterns for the most specialized species, *C. trifascialis*, with the final regression model explaining the highest proportion (20.8%) of variation in abundance for this species. The variance components analysis indicated that variation in the abundance of *C. trifascialis* among sites was as high as variation among locations, providing further support for the influence of dietary resource availability. Feeding observations conducted across five different geographical locations revealed that the that the diet of *C. trifascialis* is highly consistent among locations, whereby this species feeds predominantly on *Acropora* corals as shown previously in Lawton et al. (In press). Accordingly, the abundance of *C. trifascialis* was best predicted by a model that included the availability of *Acropora* corals rather than total dietary resource availability. In contrast, feeding observations indicated that *C. auriga*, *C. vagabundus*, *C. citrinellus*, and *C. lunulatus* were all dietary generalists, and also altered their diets among locations in response to differing availability of certain prey (Lawton et al. In press), across the five study locations. In contrast to *C. trifascialis*, the abundance of three of these species was best predicted by regression models that included indices of total dietary resource availability (coral species diversity/richness) and variation in their abundance among sites was much lower than variation among locations. However, the low proportion of variance explained by the multiple regression models for all five species indicates that the relationship between dietary resource availability and local abundance is not strong and suggests that other factors are likely to have an important influence on the local abundance of these butterflyfish.

Contrary to numerous studies that have found a strong positive relationship between total hard coral cover and butterflyfish abundance (e.g., Bell and Galzin 1984; Bouchon-Navaro et al. 1985; Cadoret et al. 1999; Bozec et al. 2005; Pratchett and Berumen 2008; Emslie et al. 2010), our results indicate that total coral cover is not an important predictor of abundance for individual butterflyfish species. There are several possible reasons why we did not find the same relationship between abundance and coral cover as these previous studies. In contrast to the current study, most previous studies have only considered the influence of coral cover on the abundance of the entire butterflyfish assemblage or specific feeding guilds (e.g., obligate corallivores). However, relationships between the abundance of individual species and coral cover are likely to vary from that of the butterflyfish assemblage due to community-level interactions. Biogeographical studies have shown that the abundance of individual butterflyfish species is negatively related to the abundance of the total butterflyfish community (Findley and Findley 2001), suggesting that factors such as interspecific competition and density compensation can strongly influence the abundance of individual species independently of total coral cover. The relationship between total coral cover and the abundance of individual butterflyfish species has only been investigated by Pratchett and Berumen (2008), who found a strong positive correlation for all obligate corallivore species at the scale of a single reef, Lizard Island. However, the total coral cover at sites sampled in their study ranged from roughly 2% to 30%. In comparison, total coral cover at sites in the current study ranged from 6% to 80%. The absence of total coral cover as a significant variable in our multiple regression models suggests that although total coral cover and abundance of individual butterflyfish appear to be linearly related at low to moderate levels of coral cover (e.g., Pratchett and Berumen 2008), the overall relationship is more likely to be asymptotic, such that further increases in coral cover above a certain threshold (e.g., 40% coral cover) have limited influence on the abundance of individual butterflyfish.

The relatively weak effect of dietary resource availability on variation in abundance of all five coral-feeding butterflyfish is also in contrast to previous studies of other coral reef fish, which have shown that that resource availability (specifically, coral cover) is a major determinant of abundance patterns for individual species at geographic spatial scales (e.g., gobies: Munday 2002; damselfish: Holbrook et al. 2000). Our contrasting findings could be a result of several factors. For some of these butterflyfish, it is possible that the resource categories we used were too poorly resolved to effectively assess variation in the abundance of key dietary components. This is likely to be the case for species such as *C. auriga*, for which small motile invertebrates comprise a significant proportion of diet (Anderson et al., 1981; Pratchett 2005). It was assumed that bites on noncoral substrates not obviously occupied by any macro-invertebrates were targeting these organisms as has been shown previously (Anderson et al. 1981). As their availability is extremely difficult to quantify in the field, our resource category of “non-coral substrates” was used as a proxy. However, the availability of noncoral substrates may not capture the true availability of small motile invertebrates. Obligate coral-feeding species, such as *C. trifascialis* and *C. lunulatus*, exhibit strong preferences for specific coral species (Pratchett 2005). Availability of these individual coral species may vary significantly from the availability of coral taxa groupings that were used in this study. Consequently, categorization of dietary resources at a finer taxonomic resolution may be necessary to reflect true dietary resource availability for these butterflyfish.

A further possibility is that availability of dietary resources is not the primary driver of local abundance at a geographic scale for these butterflyfish. The inclusion of our variable representing interspecific competition (the ratio of total coral cover to the total abundance of all congeneric butterflyfish) in final regression models indicates that competitive interactions are likely to influence local abundance. Competitive interactions may influence the range of habitats used by a species and asymmetric competition between species can lead to the exclusion of subordinate competitors from mutually preferred habitats (Connell 1983; Abramsky et al. 1990; Young 2004; Bonin et al. 2009). Although the overall predictive power of our final regression models was low, the highly significant negative correlation between our interspecific competition variable and abundance for three of the five focal species is supported by previous studies that have shown that abundance of individual species may increase in the absence of interspecific competitors (Schmitt and Holbrook 1990; Robertson 1996). Competitive interactions are likely to have the strongest influence on the local abundance of obligate coral-feeding butterflyfish as this variable was included in regression models for *C. citrinellus*, *C. lunulatus*, and *C. trifascialis—*the three species with the highest proportional consumption of hard corals. In agreement with previous observational studies of competitive interactions between butterflyfish (Crosby and Reese 2005; Berumen and Pratchett 2006a), our results suggest that defense of preferred coral resources by dominant competitors may be negatively influencing local abundance patterns of coral-feeding butterflyfish.

Larval supply and recruitment may also be a key determinant of butterflyfish abundance at large spatial scales. Both of these factors are widely acknowledged to be highly variable across both space and time in marine populations (Doherty 1991, 2002; Caley et al. 1996). Differences in adult abundance have been shown to be strongly related to prior levels of recruitment for some reef fish (Victor 1983, 1986; Holbrook et al. 2000) and variable larval supply may be contributing to the local patterns of abundance for the butterflyfish considered here (Bell et al. 1985; Pratchett and Berumen 2008). A further possibility is that both larval supply and resource availability may be constraining local abundance, as appears to be occurring in some other reef fish populations (e.g., Forrester 1995; Schmitt and Holbrook 1999; Holbrook et al. 2000), such that recruit abundance is initially determined by larval supply then consequently regulated by resource availability. Juvenile obligate coral-feeding butterflyfish are consistently found in close association with hard coral colonies in the field (Cole and Pratchett 2011) and the distribution of several species corresponds closely to the distribution of preferred coral microhabitats (Pratchett et al. 2008a). These observations suggest that the availability of coral resources for both settlement habitat and food strongly influences the abundance of juvenile coral-feeding butterflyfish and may override initial abundance patterns established at settlement (e.g., Booth 2002). Further research is necessary to determine both the influence of resource availability on the abundance and distribution of juvenile butterflyfish, and the relationship between juvenile and adult abundance.

Theory predicts that the abundance of specialist species should be lower than that of generalist counterparts, due to increased limits imposed by a restricted set of critical resources (Brown 1984). Our findings confirmed that dietary resource availability has a greater influence on the abundance of the most specialized species (*C. trifascialis*), but this species was generally more abundant, not less abundant, than generalist counterparts. This is probably due to the high abundance of *Acropora* corals at most locations. Consequently, it should not be assumed that specialists will always be rarer than generalists. *Chaetodon trifascialis* is frequently one of most abundant butterflyfish throughout its geographic range (Jones et al. 2002), and is generally only rare where there has been systematic depletion of *Acropora* corals (Berumen and Pratchett 2006b). Our results highlight the need to be wary of assigning specialized species high vulnerability status due to an assumed low abundance and resource dependence without conducting independent assessments. Supporting this, recent research has demonstrated that *C. trifascialis* has a greater level of feeding plasticity and therefore a greater capacity to respond to coral loss than previously assumed (Lawton et al. In press), while genetic evidence suggests there is a high potential for *C. trifascialis* to recover from population declines (Lawton et al. 2011). These findings indicate that the vulnerability of *C. trifascialis* to coral loss on reefs is likely to be lower than has been previously assumed.

## Appendix

**Table A1:** Proportional consumption of eight dietary categories by five butterflyfish across five locations. Data are pooled across *n* replicate feeding observations for each species of butterflyfish. Location abbreviations follow Figure 1. ACRO, *Acropora*; POR, *Porites*; MON, *Montipora*; POC, *Pocillopora*; FAV, Favidae; OHC, other hard corals; NCS, noncoral substrates; OTH, other

Location	Site	*n*	ACRO	POC	MON	POR	FAV	OHC	NCS	OTH
*C. auriga**										
HI	Blue Pools	20	0.0	0.0	0.0	0.0	0.0	0.0	99.3	0.7
	North Wistari	20	1.2	0.0	0.0	0.0	0.0	0.0	94.0	4.8
	2nd Point	21	0.0	0.0	0.0	0.0	0.0	0.0	95.8	4.2
LI	Bird Islet	20	0.0	1.6	0.0	0.0	0.0	0.0	98.4	0.0
	Lizard Head	20	0.0	2.1	0.0	0.0	0.0	0.4	96.3	1.2
	South Island	21	8.7	2.5	0.4	0.0	3.7	0.4	83.9	0.4
NC	Ilot Nge	20	6.5	2.4	0.0	0.0	0.0	0.0	86.2	4.9
	Recif Senez	20	0.7	0.0	0.2	0.0	0.0	0.0	99.1	0.0
	Seche Croissant	20	0.4	0.0	1.6	1.2	0.0	12.5	69.1	15.2
FP	Motu Ahi	20	0.0	0.0	0.0	1.0	0.0	0.0	99.0	0.0
	Temea	20	0.0	0.0	3.2	10.4	0.0	0.0	86.4	0.0
	Tiahura	20	0.0	0.0	1.0	2.1	0.0	0.0	96.9	0.0
*C. vagabundus*										
HI	Blue Pools	22	0.0	0.4	0.0	0.0	0.0	0.0	86.2	13.5
	North Wistari	22	0.6	0.3	0.0	0.3	0.0	0.3	95.7	2.8
	2nd Point	22	0.0	0.4	0.0	0.0	0.4	0.4	98.3	0.4
LI	Bird Islet	20	7.7	0.0	0.0	0.0	0.3	0.0	92.1	0.0
	Lizard Head	31	0.7	0.5	0.3	0.2	0.5	0.0	95.9	2.0
	South Island	22	1.5	0.0	0.0	0.0	2.0	1.0	94.0	1.5
PNG	Christines	20	1.7	2.8	7.6	10.4	3.8	0.0	62.5	11.1
	Lubaluba	23	2.0	10.5	3.9	7.9	0.3	2.6	70.7	2.0
	Susans	21	4.0	5.3	21.9	6.4	4.3	0.5	50.1	7.5
NC	Ilot Nge	20	9.9	9.3	2.5	0.3	0.0	2.0	71.3	4.8
	Recif Senez	20	0.0	0.0	1.3	0.0	0.0	0.0	97.1	1.6
	Seche Croissant	20	0.6	8.2	0.0	1.5	0.9	3.2	48.8	36.8
FP	Motu Ahi	21	0.0	0.5	3.9	0.5	0.0	0.0	94.7	0.5
	Temea	20	0.0	2.5	2.5	5.3	0.0	0.0	89.8	0.0
	Tiahura	20	0.0	0.0	5.0	1.7	0.0	0.0	93.3	0.0
*C. citrinellus*										
HI	Blue Pools	24	51.1	3.0	3.9	0.7	0.7	5.3	35.3	0.2
	North Wistari	22	39.8	8.4	1.1	0.4	0.0	6.9	35.8	7.6
	2nd Point	20	8.2	2.8	4.1	0.8	3.4	13.1	65.7	1.8
LI	Bird Islet	20	27.3	31.4	7.5	1.0	4.1	4.1	21.1	3.6
	Lizard Head	20	27.0	23.5	1.8	0.5	1.4	1.8	34.2	9.8
	South Island	20	24.8	30.9	5.0	0.6	6.5	7.1	21.3	3.8
PNG	Christines	21	17.3	22.5	8.6	19.1	5.0	8.4	14.5	4.5
	Lubaluba	21	18.3	5.7	6.6	5.2	5.4	0.3	57.6	0.9
	Susans	20	24.9	32.4	21.0	6.4	1.5	2.2	11.5	0.0
NC	Ilot Nge	20	40.4	17.3	1.1	1.1	3.1	0.2	36.8	0.0
	Recif Senez	20	27.3	4.6	0.2	0.0	0.0	0.0	67.2	0.7
	Seche Croissant	20	13.8	2.6	19.8	0.2	0.0	0.2	55.9	7.6
FP	Motu Ahi	20	0.5	25.1	45.7	4.8	0.0	0.2	23.7	0.0
	Temea	21	0.8	7.7	7.1	7.9	0.5	0.0	75.8	0.2
	Tiahura	20	0.7	1.6	8.4	7.1	0.2	0.4	81.6	0.0
*C. lunulatus*										
HI	Blue Pools	21	35.7	13.5	9.8	8.1	3.5	27.8	1.7	0.0
	North Wistari	24	73.3	13.3	5.9	1.3	1.2	4.7	0.3	0.0
	2nd Point	20	32.5	4.5	35.8	3.1	2.6	18.4	2.6	0.5
LI	Bird Islet	21	44.2	22.2	7.9	7.2	11.4	5.6	0.5	0.9
	Lizard Head	23	53.7	27.8	7.5	2.1	4.5	3.8	0.7	0.0
	South Island	23	56.3	25.0	3.2	5.2	6.3	3.4	0.7	0.0
PNG	Christines	21	23.7	9.2	16.4	29.1	3.8	17.0	0.0	0.8
	Lubaluba	23	8.2	1.3	9.0	31.7	8.8	39.9	0.2	0.8
	Susans	22	37.3	25.9	15.3	17.5	1.6	2.4	0.0	0.0
NC	Ilot Nge	20	36.3	35.0	9.5	14.9	1.9	1.1	1.4	0.0
	Recif Senez	20	89.5	1.6	8.6	0.0	0.0	0.0	0.3	0.0
	Seche Croissant	20	38.0	7.3	42.6	6.8	0.0	4.5	0.9	0.0
FP	Motu Ahi	21	3.0	4.9	38.5	46.2	0.0	4.3	3.0	0.0
	Temea	20	0.6	8.8	36.9	52.9	0.0	0.0	0.9	0.0
	Tiahura	20	0.0	0.3	21.9	65.6	0.5	6.2	5.7	0.0
*C. trifascialis*										
HI	Blue Pools	26	98.5	0.2	0.8	0.0	0.0	0.6	0.0	0.0
	North Wistari	26	99.1	0.4	0.0	0.0	0.5	0.0	0.0	0.0
	2nd Point	21	95.5	0.0	3.8	0.0	0.0	0.8	0.0	0.0
LI	Bird Islet	20	92.7	7.3	0.0	0.0	0.0	0.0	0.0	0.0
	Lizard Head	23	94.8	4.6	0.0	0.0	0.0	0.0	0.6	0.0
	South Island	21	90.8	9.2	0.0	0.0	0.0	0.0	0.0	0.0
PNG	Christines	21	96.7	2.4	0.3	0.2	0.0	0.3	0.0	0.0
	Lubaluba	20	91.8	7.7	0.0	0.5	0.0	0.0	0.0	0.0
	Susans	20	87.6	11.4	0.5	0.5	0.0	0.0	0.0	0.0
NC	Ilot Nge	20	98.3	0.0	0.7	0.0	0.0	1.1	0.0	0.0
	Recif Senez	20	99.6	0.4	0.0	0.0	0.0	0.0	0.0	0.0
	Seche Croissant	20	95.8	0.0	4.2	0.0	0.0	0.0	0.0	0.0
FP	Motu Ahi	20	43.2	26.7	24.8	5.3	0.0	0.0	0.0	0.0
	Temea	20	59.9	28.1	8.9	3.1	0.0	0.0	0.0	0.0
	Tiahura	20	66.6	19.9	9.7	3.0	0.0	0.8	0.0	0.0

* *C. auriga* not present at sampling sites in PNG.
